# Cardiomyopathies in 100,000 genomes project: interval evaluation improves diagnostic yield and informs strategies for ongoing gene discovery

**DOI:** 10.1186/s13073-024-01390-9

**Published:** 2024-10-29

**Authors:** Katherine S. Josephs, Eleanor G. Seaby, Philippa May, Pantazis Theotokis, Jing Yu, Avgi Andreou, Hannah Sinclair, Deborah Morris-Rosendahl, Ellen R. A. Thomas, Sarah Ennis, Angharad M. Roberts, James S. Ware

**Affiliations:** 1 National Heart and Lung Institute, Imperial College London, London, UK; 2 Royal Brompton and Harefield Hospitals, Guy’s and St Thomas’ NHS Foundation Trust, London, UK; 3 Genomic Informatics Group, Faculty of Medicine, University of Southampton, Southampton, UK; 4 Paediatric Infectious Diseases, Imperial College London, St Mary’s Hospital, London, UK; 5 Translational Genomics Group, Broad Institute of MIT and Harvard, Cambridge, MA USA; 6 Department of Immunology and Inflammation, Faculty of Medicine, Imperial College London, London, UK; 7 MRC Laboratory of Medical Sciences, Imperial College London, London, UK; 8 The Innovation Building, Novo Nordisk Research Centre Oxford, Oxford, UK; 9 SSt George’s University Hospitals NHS Foundation Trust, St George’s University of London, London, UK; 10 Guy’s and St Thomas’ NHS Foundation Trust, London, UK; 11 Genomics England, London, UK; 12 Great Ormond Street Hospital NHS Foundation Trust, London, UK

**Keywords:** Cardiomyopathy, Paediatric, Genome sequencing, 100,000 Genomes Project, Genetic diagnosis

## Abstract

**Background:**

Cardiomyopathies are clinically important conditions, with a strong genetic component. National genomic initiatives such as 100,000 Genome Project (100KGP) provide opportunity to study these rare conditions at scale beyond conventional research studies.

**Methods:**

We present the clinical and molecular characteristics of the 100KGP cohort, comparing paediatric and adult probands with diverse cardiomyopathies. We assessed the diagnostic yield and spectrum of genetic aetiologies across clinical presentations. We re-analysed existing genomic data using an updated analytical strategy (revised gene panels; unbiased analyses of de novo variants; and improved variant prioritisation strategies) to identify new causative variants in genetically unsolved children.

**Results:**

We identified 1918 individuals (1563 probands, 355 relatives) with cardiomyopathy (CM) in 100KGP. Probands, comprising 273 children and 1290 adults, were enrolled under > 55 different recruitment categories. Paediatric probands had higher rates of co-existing congenital heart disease (12%) compared to adults (0.9%). Diagnostic yield following 100KGP’s initial analysis was significantly higher for children (19%) than for adults (11%) with 11% of diagnoses overall made in genes not on the existing UK paediatric or syndromic CM panel. Our re-analysis of paediatric probands yields a potential diagnosis in 40%, identifying new probable or possible diagnoses in 49 previously unsolved paediatric cases. Structural and intronic variants accounted for 11% of all potential diagnoses in children while de novo variants were identified in 17%.

**Conclusions:**

100KGP demonstrates the benefit of genome sequencing over a standalone panel in CM. Re-analysis of paediatric CM probands allowed a significant uplift in diagnostic yield, emphasising the importance of iterative re-evaluation in genomic studies. Despite these efforts, many children with CM remain without a genetic diagnosis, highlighting the need for better gene-disease relationship curation and ongoing data sharing. The 100KGP CM cohort is likely to be useful for further gene discovery, but heterogeneous ascertainment and key technical limitations must be understood and addressed.

**Supplementary Information:**

The online version contains supplementary material available at 10.1186/s13073-024-01390-9.

## Background

Cardiomyopathies (CMs) are serious, chronic heart diseases characterised by structural and functional abnormalities of the myocardium in the absence of coronary artery disease, hypertension, valvular disease and congenital heart disease sufficient to cause the observed abnormality [1, 2]. They are highly heritable and a leading cause of heart failure and sudden cardiac death.

CMs can arise at any age: in children they are rare with population studies estimating an incidence of 1 case per 100,000 person-years, with a significant peak in infancy [3–5] and a second, smaller peak in adolescence [6]. In adults, they are much more common with prevalence estimates of around 1 in 250 for dilated cardiomyopathy (DCM) and 1 in 500 for hypertrophic cardiomyopathy (HCM) [7, 8]. These are likely underestimates and the true burden of disease and its complications are still being realised [8].

Genetic testing is now routine in the clinical investigation of many CMs [9]. A genetic diagnosis enables individualised management, informing family screening and reproductive options, and increasingly targeted therapies and interventions. However, despite significant progress in this area, most cardiomyopathy patients remain genetically unexplained. Diagnostic yields range between 20 and 60% and are dependent on age (lower in infancy) [10], cardiomyopathy type (typically lower in DCM and restrictive cardiomyopathy (RCM)) and family history (lower in the absence of familial disease) [11, 12]. Significant challenges remain in fully defining the genetic architecture, particularly for paediatric cardiomyopathy (PCM) where the number of dedicated studies is still relatively small [10, 13–19].

The 100,000 Genomes Project (100KGP), run by Genomics England (GEL), was a UK government funded research project which sequenced thousands of genetically undiagnosed rare disease patients. It offered the potential for diagnostic discovery for patients where conventional clinical testing, notably Sanger sequencing or targeted panels, did not yield a molecular diagnosis. A pilot study of all rare disease in the project showed a diagnostic yield of 25% [20]. 100KGP comprises a rich database of genotype and phenotype data made available to approved researchers. For CM, it offers the opportunity to compare adults and children recruited under the same criteria and to study the underlying genetic complexity in a relatively large number of families.

Here we seek to comprehensively describe the CM cohort in 100KGP. We explore differences between the adult and paediatric cohorts and reveal the diagnostic yield and spectrum of genes implicated after GEL’s initial analysis. Additionally, leveraging the opportunity for re-analysis afforded by genome sequencing, we seek to uplift the diagnostic yield in genetically unexplained paediatric cases. Finally, we highlight important practical considerations for other researchers working with the 100KGP cohort and other large-scale rare disease programmes.

## Methods

### 100,000 Genome project

The 100KGP offered genome sequencing to ~ 90,000 affected and unaffected participants from families with rare disease and cancer. The diagnostic workflow for the 100KGP has been previously described [20]. Briefly, patient data were analysed after application of a PanelApp [21] gene panel (or panels) selected by 100KGP based on the recruited disease category and the human phenotype ontology (HPO) terms [22] submitted by the referring clinician. Rare predicted loss of function and/or de novo variants affecting genes in the applied panels were classed as tier 1 variants; rare missense or other variant types affecting genes in the applied panels were classed as tier 2. Other filtered variants not in genes on the applied panels were designated as tier 3. Routinely, variants in tier 1 and tier 2 were assessed by clinical scientists in National Health Service (NHS) accredited laboratories and reports were returned to patients.

Inclusion criteria for CM in 100KGP required prior testing of specific genes depending on the disease [23]. Initially all cases, unless presenting with HCM < 40 years, had to have at least one other affected family member. These criteria were later relaxed which allowed those without prior genetic testing or a family history to also be recruited. The relaxation of eligibility criteria was not standardised across recruitment centres and occurred throughout the timeline of the project.

Participants recruited under CM were assigned a high-level category by 100KGP based on their disease called the ‘normalised specific disease’. This category was one basis for gene panel selection by GEL. For CMs, the categories included hypertrophic cardiomyopathy, dilated cardiomyopathy, dilated cardiomyopathy and conduction defects, arrhythmogenic right ventricular cardiomyopathy, restrictive cardiomyopathy and left ventricular non-compaction cardiomyopathy. There was no specific category for paediatric cardiomyopathy. Additional phenotype data in the Genomics England Research Environment (RE) are stored as HPO [22] terms in MySQL databases within a LabKey data management system. Participants were labelled as the proband or another family member and as affected or unaffected within a family. Age at diagnosis was recorded. In addition, probands were labelled as ‘complete’ if a report confirming either a genetic diagnosis or a negative result had been returned to the recruiting Genomic Medicine Centre (GMC) and as ‘solved’ if they had a genetic diagnosis that explained their presenting disease.

### Data access

We obtained access to the RE and high-performance cluster through membership of Genomics England Clinical Interpretation Partnerships: *Quantitative methods, machine learning, and functional genomics* and *Cardiovascular* domains. This provided access to genome sequencing and phenotype data for 90,190 individuals sequenced as part of the 100,000 Genomes Project. All participants gave written informed consent.

### Identifying patients with cardiomyopathies

We queried 90,190 individuals (affected individuals and unaffected relatives) with genome sequencing data (main-programme_v16_2022-10–13). We searched for any participant recruited under CM as per their ‘normalised specific disease’. Participants were recruited under hypertrophic cardiomyopathy, dilated cardiomyopathy, dilated cardiomyopathy and conduction defects, arrhythmogenic right ventricular cardiomyopathy and left ventricular non-compaction cardiomyopathy. No participants were recruited specifically under restrictive cardiomyopathy. We designated these participants presenting with CM as a principal feature as ‘Primary CM’. We next searched for any participant with ‘cardiomyopathy’ contained within the free text of any HPO term, e.g. dilated cardiomyopathy or hypertrophic cardiomyopathy. All those participants who were not already labelled as ‘Primary CM’ were designated as ‘Complex CM’. Complex CM includes participants with CM and an associated syndrome or rare disease, e.g. Noonan syndrome or Duchenne muscular dystrophy or those with CM recruited under a different disease category ostensibly not directly related, e.g. chronic kidney disease or hearing loss. We recognise this grouping is imperfect, that some Primary CM participants may have additional phenotypes and that Complex CM will be very heterogeneous, however, it allows separation of those recruited specifically under CM and those who were not.

### Defining the age of participants with cardiomyopathy

Age of onset is not reliably recorded for participants in 100KGP; ‘0’ was the default value in the dataset, and consequently can represent infant-onset or missing data. Therefore, we defined a participant as a child when any of the following criteria were met: age at consent ≤ 16 years old; age at diagnosis ≤ 16 years old; age at onset between 1 and 16 years inclusive; age at first cardiology appointment (using hospital episode statistics (HES) outpatient codes 320 or 170) ≤ 16 years old. Participants with an age ≤ 16 years old documented at the time of consent to participate were designated as children meeting stringent age criteria.

Demographic, phenotype and genotype data for all participants labelled as Primary or Complex CM were queried and collated by joining LabKey tables. Mortality data is imported by GEL from NHS England and was accessed through the relevant LabKey table, main-programme_v18_2023-10–13. Survival time from disease onset was calculated using time from ‘date of onset’ to either ‘date of death’ or ‘date of last data import’ in the LabKey mortality table. If age of onset was recorded as ‘0’ or otherwise missing, ‘date of diagnosis’ was used instead of ‘date of onset’. Median follow-up time for adults from date of onset was 115 months (maximum 785 months). Median follow-up time for children from date of onset was 88 months (maximum 787 months).

### Reanalysis of genetically unsolved paediatric probands

**Fig. 1:**
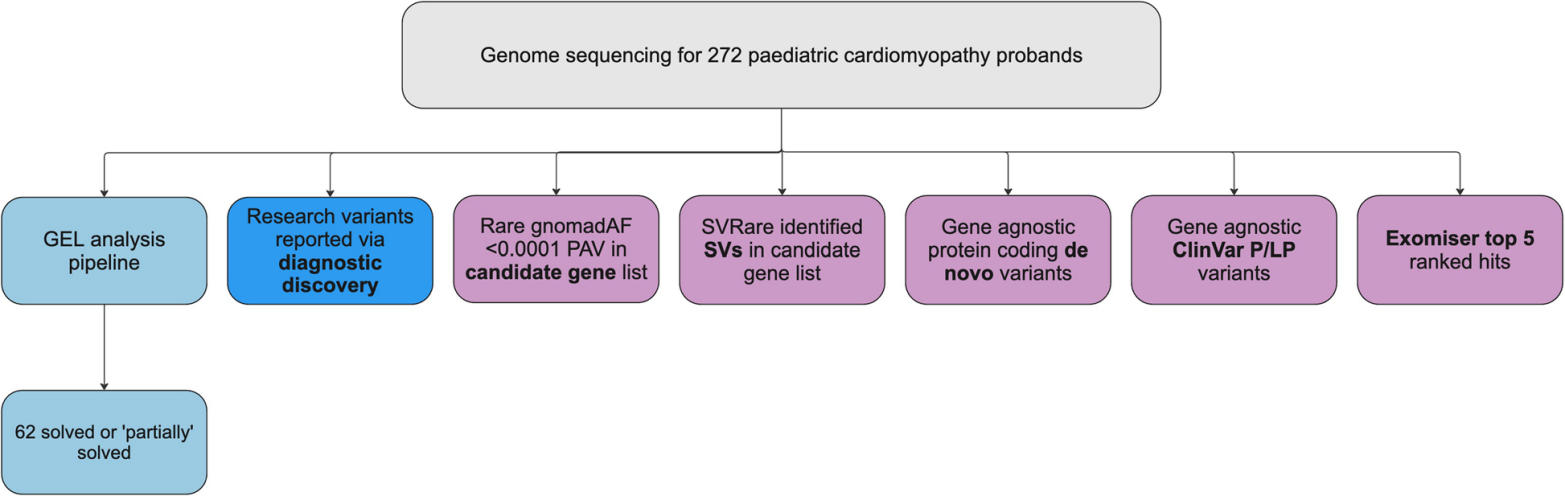
Overview of analyses and sources of diagnoses for 272 paediatric cardiomyopathy probands with available genome sequencing data in 100,000 Genomes Project. Initial GEL analysis pipeline identified (partial) diagnoses for 62 probands; other potentially causative variants were identified by other researchers accessing the 100KGP data and reported via the diagnostic discovery pathway. In addition, we prioritised variants by identifying rare protein altering variants in a candidate gene list; identifying structural variants in a candidate gene list; reviewing all protein coding de novo variants where trio data was available; reviewing variants flagged as pathogenic or likely pathogenic by ClinVar; and reviewing top 5 Exomiser hits. GEL, Genomics England; P/LP, pathogenic/likely pathogenic; PAV, protein altering variant; SV, structural variant

We carried out further analysis on the unsolved PCM cohort (see Fig. 1). Sequencing, alignment and variant calling was carried out by GEL’s initial data processing pipeline [20]. Variants aligned to GRCh37 were lifted over to GRCh38 using GATK LiftoverVcf (GATK version 4.2.2.0). Individual VCF files were then merged using bcftools merge (BCFtools version 1.16) and annotated with Ensembl Variant Effect Predictor [24] (VEP) version 109. Rare (gnomAD [25] v3 exome allele frequency < 0.0001), protein altering variants in nuclear-encoded genes from a candidate list (see below) were extracted and analysed. In addition, ClinVar pathogenic (P) or likely pathogenic (LP) variants (no conflicts) (ClinVar version 2023-08-19), relevant to the patient’s phenotype, were extracted and analysed; details of the top 5 ranked Exomiser [26] hits for each proband were extracted from the GEL exomiser LabKey table (main-programme_v18_2023-10–13); and de novo and structural variants were also analysed (see below for details).

### Candidate gene list

**Fig. 2:**
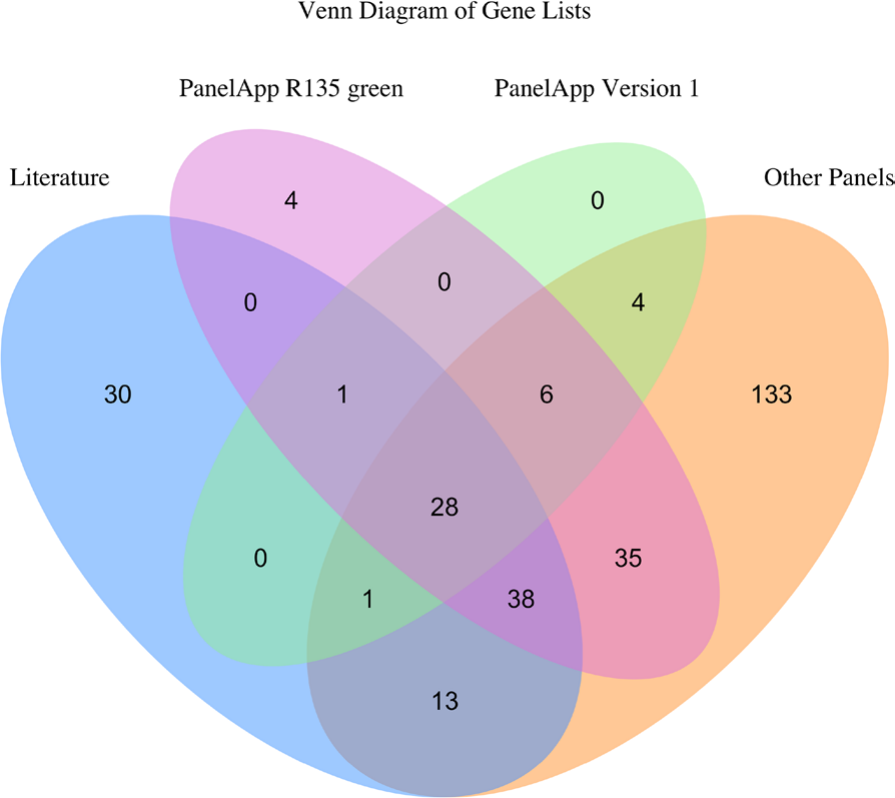
Venn diagram of genes associated with paediatric cardiomyopathy. Literature—genes identified in paediatric cardiomyopathy studies and case reports; PanelApp R135 green—genes on R135 paediatric or syndromic cardiomyopathy PanelApp panel v3.44 with the highest level of evidence; PanelApp Version 1—genes on the original versions of hypertrophic, dilated, left ventricular non-compaction and arrhythmogenic cardiomyopathy panels most likely applied to those recruited to 100,000 Genomes Project in 2015–2018; Other Panels—genes on 6 other paediatric cardiomyopathy panels including Invitae, GeneDx, Ambry, Laboratory for Molecular Medicine, PanelApp Australia and Amsterdam Genetics. See Additional file 1: Tables S1–S4 for full gene lists

All designated ‘green’ (highest level of confidence for gene-disease association) and ‘amber’ (moderate level of confidence for gene-disease association) nuclear-encoded genes on the PanelApp paediatric or syndromic cardiomyopathy panel R135 v.3.44 [21] (R135 CM panel) were included. In addition, nuclear-encoded genes from six other PCM panels and genes identified as potentially disease causing from previous PCM studies were included. See Fig. 2 and Additional file 1: Tables S1–S4 for full list and references.

### Protein coding de novo variant analysis

One hundred thirty-four out of 273 paediatric probands were recruited as trios and have genomic data available for both parents. We analysed de novo variants fulfilling GEL’s stringent criteria (see Additional file 1: Table S5 for details). Maternity and paternity are confirmed (not presumed). Variants were extracted from the LabKey table: denovo_flagged_variants, main-programme_v16_2022-10–13. Variants aligned to GRCh37 were lifted over to GRCh38 using GATK LiftoverVcf (GATK version 4.2.2.0) and then annotated using VEP version 109. Variants were then filtered to exclude non-coding VEP consequence terms.

### Structural variant analysis

Structural variants defined as DNA changes that extend to at least 50 nucleotides were identified in the paediatric probands using SVRare [27]. This uses a database of 554,060,126 structural variants (SVs) called by Manta [28] and Canvas [29] from the 71,408 rare disease participants in 100KGP. SV types called by Manta include deletions, duplications, inversions, insertions and duplications of tandem repeats but only deletions, duplications and inversions were considered in SVRare; SV types called by Canvas include LOSS and GAIN, both considered in SVRare. We prioritised rare SVs (≤ 5 database calls) that overlapped the coding regions of one or more of our candidate genes. SVs were inspected manually using BAM files in the Integrative Genomics Browser (IGV).

### Diagnostic discovery pathway

Over time, approved researchers have accessed 100KGP data in the RE and identified potential missed diagnoses or novel discoveries. Such findings are shared with GEL through their diagnostic discovery pathway, whereby candidate variants are assessed by a multidisciplinary team and, where appropriate, sent to diagnostic laboratories for variant classification. Variants passed through the diagnostic discovery pathway are stored in a LabKey table in the RE and were queried for any related to the paediatric CM cohort (main-programme_v18_2023-10–13).

### Variant classification

For cases labelled as solved, the GEL variant classification was used. During the re-analysis of the PCM cohort, variants prioritised as potentially disease-causing or contributing to the proband’s phenotype were classified according to the original framework set out by the American College of Medical Genetics and Genomics and the Association for Molecular Pathology [30] (ACMG guidelines), with modifications for variant type [31], gene/disease [32], UK guidelines [33] and scientific judgement, as appropriate. For ACMG classification, candidate genes that were not part of the PanelApp panel were treated as having confirmed disease-gene association if there was independent evidence of CM being part of the phenotypic spectrum associated with that gene.

### Statistical analysis

Categorical variables are summarised with frequencies by age of onset (paediatric or adult).

Chi-squared test was used to assess associations between categorical variables and age of onset (paediatric or adult). Two sample proportion tests were performed to assess differences between the proportion of overall paediatric probands and solved paediatric probands recruited as trios. Tests were two-sided and a significance level of 0.05 was used for all comparisons. A survival analysis was performed using the Kaplan–Meier method with p values derived from a log rank test. Data analysis was carried out using R v.4.0.3 and LibreOffice v.5.3.6.1 in the GEL research environment.

## Results

### Section 1: demographic and clinical characteristics of adult and paediatric cardiomyopathy in 100KGP

**Fig. 3:**
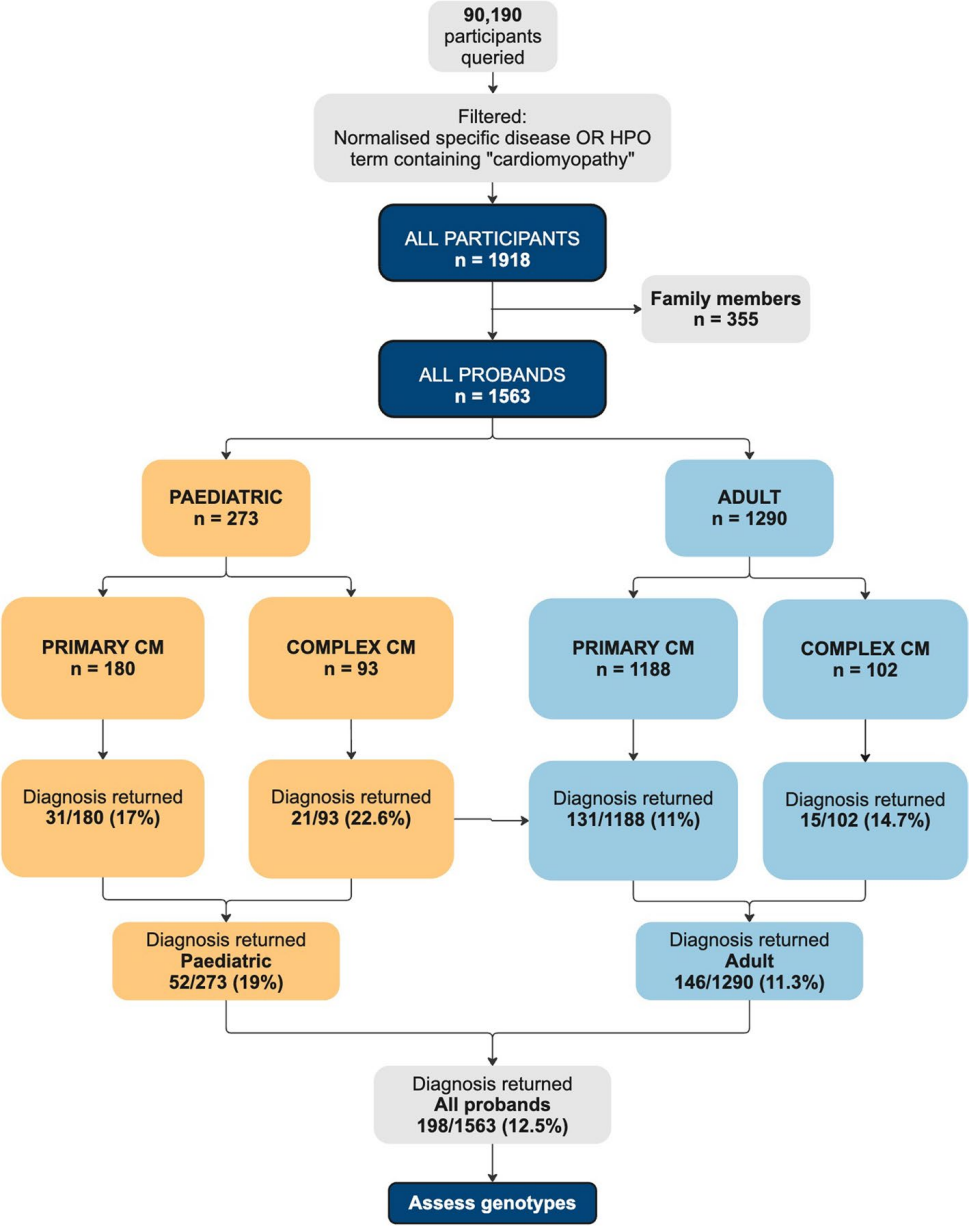
Workflow depicting the number of participants in 100,000 Genome Project (100KGP) queried and filtered for cardiomyopathy (main-programme_v16_2022-10–13). Participants were defined as paediatric if they met any of the following criteria: age at consent ≤ 16 years old; age at diagnosis ≤ 16 years old; age at onset between 1 and 16 years; age at first cardiology appointment (using hospital episode statistics (HES) outpatient codes 320 or 170) ≤ 16 years old. Primary CM: participants recruited under a specific cardiomyopathy disease category. Complex CM: participants recruited under a non-cardiomyopathy disease category with a human phenotype ontology (HPO) term containing ‘cardiomyopathy’. Diagnosis returned represents the number of probands designated as solved in 100KGP. CM, cardiomyopathy

Characteristics of the cohort are summarised in Fig. 3 and Table 1. We identified 1918 individuals of all ages with cardiomyopathies from 90,109 participants (2%) as of October 2022. The 1563 probands comprise 1290 adults (83%) and 273 children (17%). One hundred forty-four (9%) had an age at consent ≤ 16 years and were designated as children meeting stringent criteria. Overall, 1368 (88%) probands were recruited under a CM specific disease category and were designated as Primary CM, and 195 (12%) probands were recruited under another category but had an HPO term containing ‘cardiomyopathy’ and were designated as Complex CM.

**Table 1:** Demographic characteristics of 1563 cardiomyopathy probands in the 100,000 Genome Project (100KGP)

		**All probands (*n* = 1563)**	**Paediatric probands (*n* = 273)**	**Adult probands (*n* = 1290)**
		*n* (%)	*n* (%)	*n* (%)
Sex	Female	589 (38)	115 (42)	474 (37)
	Male	974 (62)	158 (58)	816 (63)
Ancestry (self-reported)	Asian	105 (7)	32 (14^a^)	73 (7^a^)
	Black	53 (3)	10 (4^a^)	43 (4^a^)
	White	1070 (68)	180 (77^a^)	890 (85^a^)
	Mixed	21 (1)	< 10	< 20
	Other	30 (2)	< 10	< 30
	Not stated	284 (18)	39 (14)	245 (19)
Family history	Yes	659 (42)	95 (35)	564 (44)
	No	904 (58)	178 (65)	726 (56)
Primary CM	ARVC	126 (8)	9 (3)	117 (9)
	DCM	383 (25)	54 (20)	329 (26)
	HCM	794 (51)	92 (34)	702 (54)
	LVNC	50 (3)	19 (7)	31 (2)
	Mixed_CM	< 20	< 10	< 10
	Mixed_other	< 20	< 10	< 10
Complex CM		195 (12)	93 (34)	102 (8)

Primary CM: probands recruited under a specific cardiomyopathy disease category

Complex CM: probands recruited under a non-cardiomyopathy disease category with a human phenotype ontology (HPO) term containing ‘cardiomyopathy’

Counts <5 must be masked when exporting data from the 100KGP research environment. Smaller values are therefore given as ranges, e.g. <5, and percentages in these instances are not reported

Family history is recorded as ‘Yes’ if either a parent or a sibling is recorded as affected with the same condition as the proband

*ARVC* Arrhythmogenic right ventricular cardiomyopathy, *DCM* Dilated cardiomyopathy, *HCM* Hypertrophic cardiomyopathy, *LVNC* Left ventricular non-compaction cardiomyopathy, Mixed_CM, probands recruited under more than one cardiomyopathy specific category, e.g. hypertrophic cardiomyopathy and left ventricular non-compaction cardiomyopathy, Mixed_other, participants recruited under a cardiomyopathy disease category and at least one other non-cardiomyopathy category, e.g. hypertrophic cardiomyopathy and corneal abnormalities

^a^% of known self-reported ancestry, excluding those where ancestry is not stated

#### Comparing adult and paediatric cardiomyopathy—demographics

Cardiomyopathies in children are much rarer than in adults, and previous work has suggested they may have a distinct genetic architecture. A proportion present almost uniquely in childhood particularly during infancy, and a proportion represent early presentations of CMs that can have a broad age of onset (seen in particular in adolescents). Given this distinction, we sought to separately characterise and compare the adult and paediatric groups. Male probands predominate across all ages: 58% of children with CM and 63% of adults. This contrasts with the broader 100KGP pilot study on all rare disease which found a higher proportion of males to females in paediatric but not adult rare disease probands [20]. Ancestry (self-reported) is not stated for a large proportion 284/1563 (18%). Excluding those where it is not stated, a greater proportion of adults (85%) than children (77%) are recorded as White, *p* = 0.0028. Asian probands make up 14% of the paediatric CM cohort and only 7% of the adult, *p* = 0.0012; this is in keeping with findings from the overall rare disease pilot [20]. Ancestry by diagnosis also differed. Notably, Asian ancestry comprises 14% of the overall paediatric cohort but 23.8% of children with ‘Complex CM’. Family history was reported in more adults than children, 44% vs 35% (see Table 1). This difference is more marked when stringent criteria were used to define children (see Additional file 1: Table S6). Those without a family history were more likely to be recruited as a trio with mother and father compared to those with a family history, *p* < 0.0001.

#### Comparing adult and paediatric cardiomyopathy—family structure, recruitment and natural history

**Fig. 4:**
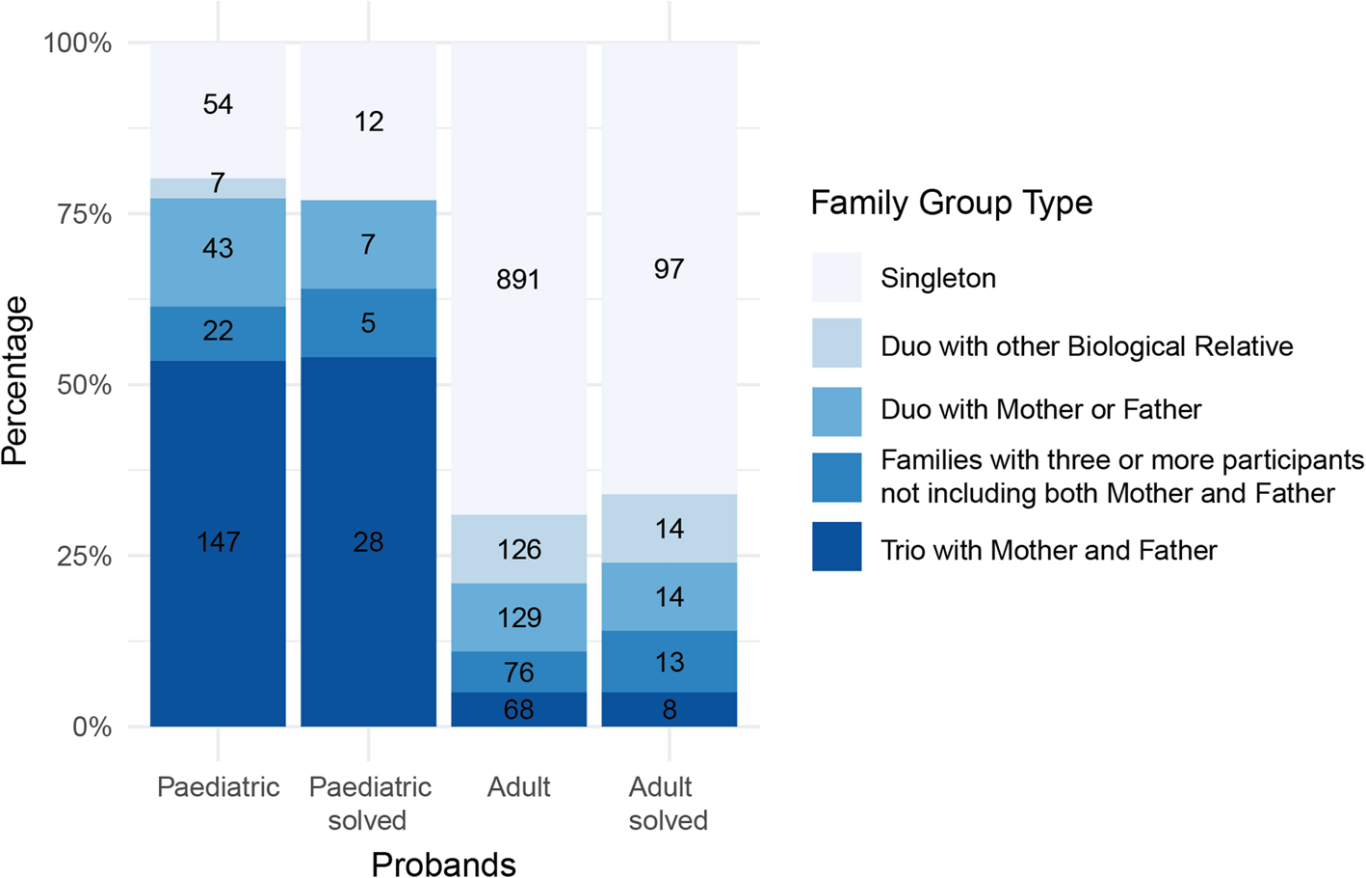
Percentages of different family structures at enrolment for paediatric and adult cardiomyopathy probands and ‘solved’ probands in 100,000 Genome Project (100KGP). Stacked bars are labelled with numbers of probands. Paediatric—paediatric probands with cardiomyopathy (CM); Paediatric solved—paediatric probands with CM and a molecular diagnosis; Adult—adult probands with CM; Adult solved—adult probands with CM and a molecular diagnosis. Singleton refers to a proband for whom no other family member was recruited, duo with other biological relative refers to a proband–non-parent pair, duo with mother or father refers to a proband-parent pair, families with three or more participants refers to a proband recruited with 3 or more biological relatives (but not both mother and father), and trio with mother and father refers to a proband and both parents ± other individuals from the family. Participants are labelled as ‘solved’ in 100KGP if they had a genetic diagnosis that explained their presenting disease

As expected, many more children were enrolled as trios, 54%, compared to 5% of adults (see Fig. 4). Most adults were recruited as singletons (69%). When paediatric probands are defined using stringent criteria, the percentage enrolled as trios is even higher (75%). Interestingly trios did not contribute more to the solved cases than unsolved cases, *p* = 0.83, and this stands when using stringent criteria to define children (see Additional file 1: Table S8).

#### Recruitment

A much larger proportion of children are grouped under Complex CM compared to adults, 34% vs 8% respectively. The proportion of Complex CM is even higher amongst children meeting stringent age criteria, 65/144 (45%) (see Additional file 1: Table S6). Those with Complex CM were recruited under a broad list comprising 52 different disease categories (see Additional file 1: Table S9). The most frequently used at any age was ‘Mitochondrial’.

#### Co-existing congenital heart disease (CHD)

Co-existing CHD was documented in 32/273 children (11.7%). Twenty-one (7.7%) had an atrial septal and/or ventricular septal defect. Eleven (4%) had another congenital heart lesion (including but not limited to aortic valve stenosis, tetralogy of Fallot, interrupted aortic arch and transposition of the great arteries). Over half of the children (17/32) with co-existing CHD were recruited under a specific CM disease category (designated as Primary CM). In comparison, only 12/1290 (0.9%) of adults had a documented CHD, the majority with atrial septal and/or ventricular septal defects.

#### Natural history: 100KGP cardiomyopathy cohort have severe disease

Amongst children, 38/273 (14%) have died. This included 15 children designated as Primary CM (15/180, 8%) and 23 with Complex CM (23/93, 25%). Nearly half of these children were recruited with an undiagnosed metabolic condition or a mitochondrial disorder, 17/38 (45%). The majority of children who died did so before the age of 5 years, 27/38 (71%). The median follow-up time from diagnosis was 88 months (maximum 787 months).

For adults, 126/1290 (9.8%) have died. This includes 102 adults designated as Primary CM (102/1188, 8.6%) and 24 as Complex CM (24/102, 23.5%). Over half of the adult deaths occurred in individuals recruited under HCM, 67/126 (53%). The majority of adults who died were over the age of 50, 103/126 (81.7%). The median follow-up time from diagnosis was 115 months (maximum 785 months).

**Fig. 5:**
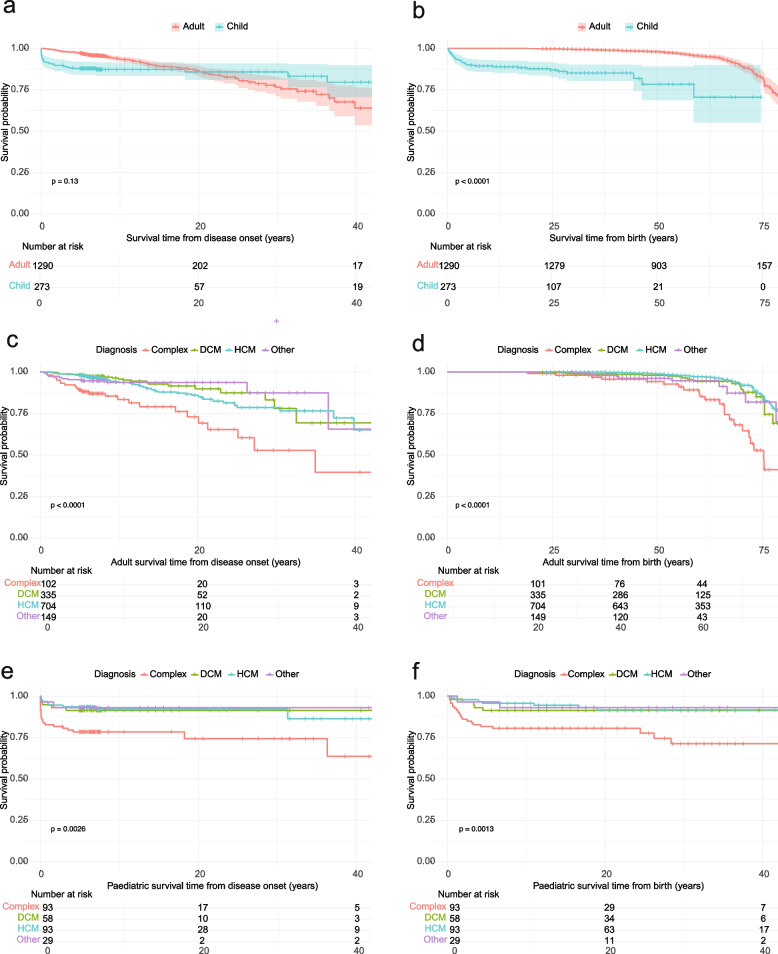
Kaplan–Meier survival analysis for adult and paediatric probands with cardiomyopathy (CM) in 100,000 Genomes Project. **a** and **b** Comparing adults and children from diagnosis and birth respectively. **c** and **d** Comparing survival probability for adults with different CM diagnoses from diagnosis (**c**) and birth (**d**). **e** and **f** Comparing survival probability for children with different CM diagnoses from diagnosis (**e**) and birth (**f**). *p* values derived from a log rank test. DCM—dilated cardiomyopathy (probands recruited under DCM and any Mixed_CM or Mixed_other probands where at least 1 recruitment category was DCM); HCM—hypertrophic cardiomyopathy (probands recruited under HCM and any previous Mixed_CM or Mixed_other where at least 1 recruitment category was HCM); Other includes probands recruited under LVNC—left ventricular non-compaction cardiomyopathy or ARVC—arrhythmogenic right ventricular cardiomyopathy. Complex CM: probands recruited under a non-cardiomyopathy disease category, but with a human phenotype ontology (HPO) term containing ‘cardiomyopathy’

There was no significant difference overall between adults and children recruited to 100KGP in survival probability from the time of disease onset (see Fig. 5a). However, for children, the curve illustrates a steep drop off in survival probability at diagnosis before levelling off. This is also evident when comparing adults and children from birth (Fig. 5b). Survival probability is impacted by CM type. Those presenting with ‘Complex CM’ had the lowest survival probability (see Fig. 5c–f). These survival probabilities reflect only the patients recruited to the 100KGP and do not account for those who may have died before recruitment or those who were not recruited for any other reason.

### Section 2: genetic architecture of adult and paediatric cardiomyopathy in 100KGP

#### Diagnostic yield following GEL’s initial analysis

After GEL’s initial analysis, the rate of genetic diagnosis was significantly lower in adults with CM than in children (11% vs 19%, *p* = 0.0007). The solved rate for children remained at ~ 20% when using stringent criteria to define age (see Additional file 1: Table S8). An additional 10 children and 27 adults were reported as ‘partially solved’. Full variant details can be found in Additional file 1: Tables S10 and S11.

#### Diagnostic yield varies by age and cardiomyopathy subtype

The diagnostic yield was highest for those children designated as Complex CM, 23%. For children recruited specifically under HCM, it was 18% and under DCM, 17%. This is lower than other studies of paediatric CM [10, 13] reflecting what should be a discovery cohort depleted for known causes of CM.

Adults with DCM had a higher diagnostic yield (18%) compared to those with HCM (9%) reflecting the lower diagnostic yield of NHS testing for DCM vs HCM at the time (see Table 2).

**Table 2:** Number of paediatric and adult cardiomyopathy probands by disease recruitment category in the 100,000 Genome Project (100KGP) and the number and percentage of solved probands

Disease recruitment category		Paediatric	Paediatric solved	Adult	Adult solved
		Probands (*n*)	Number solved (%)	Probands (*n*)	Number solved (%)
Primary CM	DCM	58	9 (17)	335	59 (18)
	HCM	93	17 (18)	704	65 (9)
	Other	29	5 (17)	149	7 (5)
	All	180	31 (17)	1188	131 (11)
Complex CM		93	21 (23)	102	15 (15)
Total		273	52 (19)	1290	146 (11)

Primary CM: probands recruited under a specific cardiomyopathy disease category

DCM—dilated cardiomyopathy and any Mixed_CM or Mixed_other probands where at least 1 recruitment category was DCM; HCM—hypertrophic cardiomyopathy and any previous Mixed_CM or Mixed_other where at least 1 recruitment category was HCM; Other includes probands recruited under LVNC—left ventricular non-compaction cardiomyopathy or ARVC—arrhythmogenic right ventricular cardiomyopathy

Complex CM: probands recruited under a non-cardiomyopathy disease category, but with a human phenotype ontology (HPO) term containing ‘cardiomyopathy’. Participants were labelled as ‘solved’ in 100KGP if they had a genetic diagnosis that explained their presenting disease

#### Most diagnoses are attributed to known cardiomyopathy genes

**Fig. 6:**
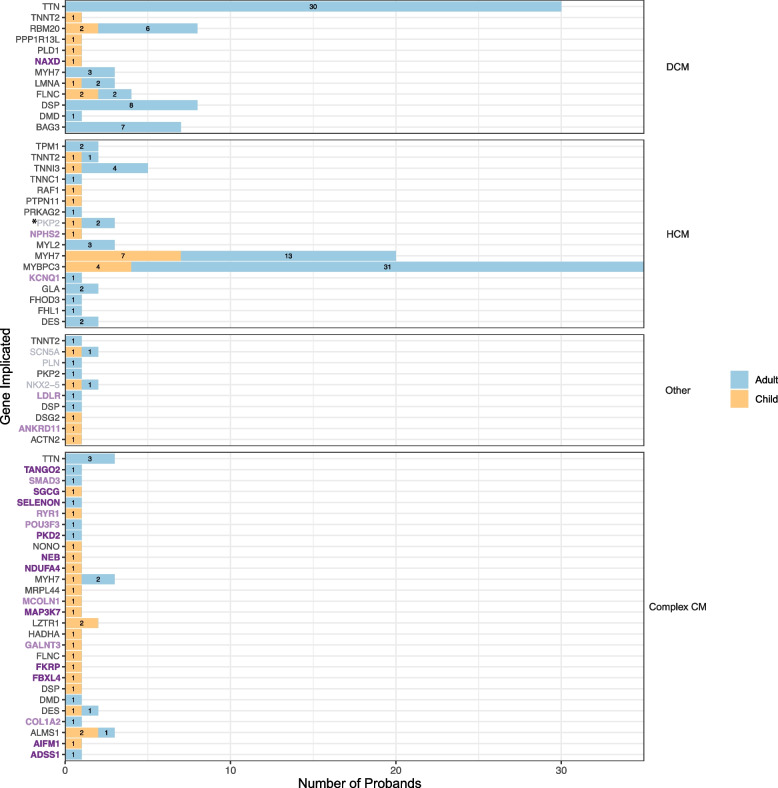
Genes implicated in the solved paediatric (*n* = 52) and adult (*n* = 146) cardiomyopathy probands in 100,000 Genome Project split by recruitment disease categories: Primary—DCM, HCM, Other (which includes ARVC and LVNC) and Complex CM (participants recruited under a non-cardiomyopathy disease category but who have a human phenotype ontology (HPO) term containing ‘cardiomyopathy’). Labels on bars indicate number of probands. Genes are colour coded: black genes are on the current R135 CM panel ‘green’ list; grey genes are on the R135 CM panel ‘green’ list but are not robustly associated with the type of cardiomyopathy reported in the solved case, e.g. *PKP2 *and HCM; dark purple genes are not on the current R135 CM panel ‘green’ list but CM is a recognised feature of the associated disease (*NDUFA4* and *FKRP* are on the ‘amber’ list); light purple genes in bold are not on the current R135 CM panel ‘green’ list and CM is not a recognised feature of the associated disease. Full variant details can be found in Tables S10 and S11. *One adult proband recruited under HCM has both a pathogenic variant in *PKP2* and a likely pathogenic variant in *LZTR1* (see Table S11 for details). R135 CM panel—NHS Genomic Medicine Service paediatric or syndromic cardiomyopathy panel (R135 v3.44); ‘green’ list—genes with the highest level of evidence; ‘amber’ list—genes with moderate evidence; ARVC, arrhythmogenic right ventricular cardiomyopathy; CM, cardiomyopathy; DCM, dilated cardiomyopathy; HCM, hypertrophic cardiomyopathy; LVNC, left ventricular non-compaction cardiomyopathy

Despite eligibility criteria requiring participants to have undergone standard genetic testing, most positive findings involve known genes that are expected to be analysed in routine diagnostic panel sequencing. For example, variants in *MYBPC3* and *MYH7* are still the most frequent cause of HCM in both adults and children (see Fig. 6).

#### Is solved really solved? 11% of CM diagnoses in 100KGP involve genes not on an existing UK CM panel

Twenty-two CM cases where the recruiting GMC concluded the case to be solved involve genes not on the current R135 CM panel ‘green’ list (see Fig. 6 genes in bold). Most of these genes (12/22) are associated with syndromic conditions where CM can be a feature but is unlikely to be found in isolation, e.g. NEB related nemaline myopathy. In keeping with this, it is mostly those with complex CM, i.e. those who were recruited under a different disease category who have findings in these genes. Variants in these genes would have been tiered by GEL for analysis because the patient’s additional phenotype terms triggered other disease panels to be applied (see dark purple genes in Table 3). Two of these twelve genes, *NDUFA4* and *FKRP*, are already on the R135 CM panel ‘amber’ list. However, going forward it would be reasonable to include the other 10 genes on a syndromic paediatric CM panel and they may already be on more comprehensive gene lists used by laboratories outside of the UK.

**Table 3:**
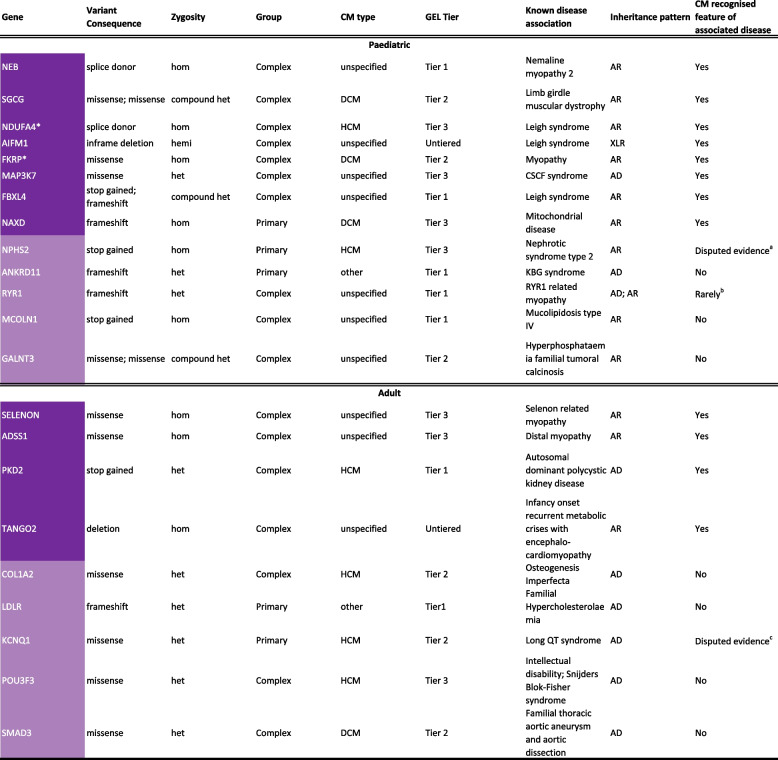
Disease and cardiomyopathy association for twenty-two genes not on the R135 CM panel ‘green’ list where variants were identified and considered diagnostic for cardiomyopathy probands in 100,000 Genomes Project (100KGP)

Left panel - variant details, the phenotype of the proband in 100KGP and how the variant was tiered in GEL's initial analysis

Right panel - known gene disease association, inheritance pattern and whether cardiomyopathy is a recognised feature of the associated disease

Dark purple genes have an associated disease where CM is a recognised feature; light purple genes have an associated disease where CM is not a recognised feature. GEL tier—all variants were tiered on GEL’s initial analysis. Briefly, tier 1: rare protein damaging variants in genes on selected panel(s); tier 2: rare protein altering variants in genes on selected panel(s); tier 3: rare protein altering variants in genes not on selected panel(s). Therefore, if a diagnosis is made in a tier 3 or untiered variant, it suggests the correct panels were not triggered by the participant’s recruitment category or phenotype and the variant(s) was prioritised for another reason, e.g. it was de novo. Additional information about why a variant was prioritised in a particular patient is not always provided in GEL

*AD* Autosomal dominant, *AR* Autosomal recessive, *CM* Cardiomyopathy, *CSCF* Cardiospondylocarpofacial, *GEL* Genomics England, *het* heterozygous, *hom* homozygous, *hemi* hemizygous, *R135 CM panel*, NHS Genomic Medicine Service paediatric or syndromic cardiomyopathy panel (R135 v3.44), *XLR* X-linked recessive

*FKRP and NDUFA4 are on the R135 CM panel ‘amber’ list (genes with moderate evidence)

^a^Cardiac abnormalities, including left ventricular hypertrophy, have been found in association with the variant p.Arg138* [34], but these findings have not been identified in patients carrying other variants [35]

^b^Cardiac involvement mainly reported in the recessive form; there is a report of adult onset HCM in the dominant form. [36, 37]

^c^As per ClinGen, all known HCM cases with *KCNQ1* variants also have another variant in, e.g. *MYH7* or *MYBPC3* that are more likely to be causing the phenotype [38, 39]

In contrast, for 10/22 genes responsible for solved cases and not on the R135 CM panel, CM is only very rarely, or not known to be associated, e.g. ANKRD11 related KBG syndrome or LDLR related familial hypercholesterolaemia (see Table 3). It is not possible from the information available in 100KGP to be sure why the recruiting centres concluded these CM cases were solved. These could be partial diagnoses where the CM phenotype remains unexplained, or the CM could be a secondary finding. In some instances, the reported CM could be an expansion of the known phenotype. Further input from the recruiting clinical centre will be needed to resolve these cases.

There are also cases documented as solved where the gene is on the CM panel, but not robustly associated with the type of CM reported, e.g. *PKP2* seen in HCM (see grey genes in Fig. 6). Again, further input regarding the phenotype of the patient is needed to unravel these cases.

#### How does the diagnostic yield compare with other rare disease in 100KGP?

As of October 2022, 17% of all rare disease participants in GEL were reported as ‘solved’—i.e. they have a genetic diagnosis that explains their presenting disease. In comparison, 12.5% of the CM cohort were reported as solved.

### Section 3: re-analysis of the paediatric cardiomyopathy probands—can we improve diagnostic yield?

**Fig. 7:**
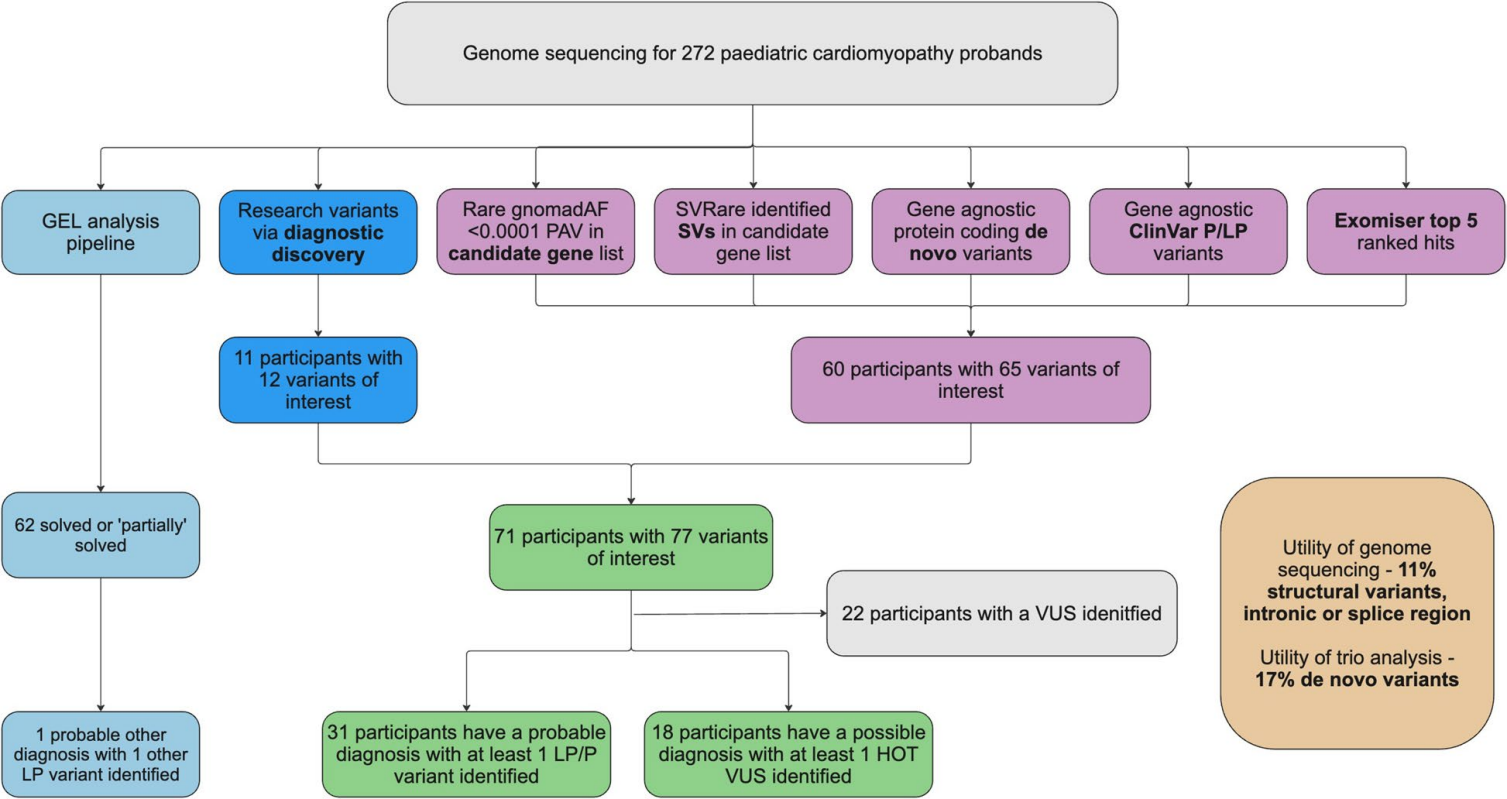
Overview of analyses and sources of diagnoses for 272 paediatric cardiomyopathy probands with available genome sequencing data in 100,000 Genomes Project. Initial GEL analysis pipeline identified (partial) diagnoses for 62 probands; 11 probands had variants identified by other researchers and reported via the Genomics England diagnostic discovery pathway. In our re-analysis, 60 probands had 65 variants of interest identified by 5 methods: identifying rare protein altering variants in nuclear-encoded genes in a candidate list; identifying structural variants in a candidate gene list; reviewing all protein coding de novo variants where trio data was available; reviewing variants flagged as pathogenic or likely pathogenic by ClinVar; reviewing top 5 Exomiser hits. GEL, Genomics England; P/LP, pathogenic/likely pathogenic; PAV, protein altering variant; SV, structural variant; VUS, variant of uncertain significance

We re-examined 210 unsolved PCM probands using the methods outlined in Fig. 7. In addition, we analysed any paediatric proband with a solved or partially solved label where their CM did not appear to be fully explained.

We identified a probable diagnosis (at least one pathogenic or likely pathogenic (P/LP) variant) in 31 unsolved PCM probands and a possible diagnosis (at least one Hot VUS) in a further 18 individuals. A classification of ‘Hot VUS’ was assigned when there was evidence towards pathogenicity (but insufficient to reach LP) and there is a reasonable likelihood that additional evidence such as RNA splicing studies, functional assays or co-segregation with disease in multiple affected family members would clarify its significance. An additional LP variant has been identified in one previously solved proband (see Table 4). No additional potentially causative variants were found for those participants labelled as ‘partially solved’ by GEL. Further variant details are given in Additional file 1: Table S12.

**Table 4:** Probable and possible diagnoses identified from the re-analysis of paediatric cardiomyopathy probands in 100,000 Genomes Project. Probable diagnosis—at least one pathogenic or likely pathogenic (P/LP) variant identified; possible diagnosis—at least one Hot VUS identified. A classification of ‘Hot VUS’ was assigned when there was evidence towards pathogenicity but it was insufficient to reach LP and there is hope that additional evidence such as RNA splicing studies, functional assays or co-segregation with disease in multiple affected family members would clarify its significance. One previously solved proband has a LP and VUS in *ALPK3* identified. The majority of variants were identified using a candidate cardiomyopathy panel (CM panel). Variants were also sourced using ClinVar, Exomiser top 5 hits and reviewing structural variants (SV) and de novo variants. Some variants were also identified by the Genomics England (GEL) diagnostic discovery pathway (diag) or identified by GEL on a second analysis. *HGVSc*, Human Genome Variation Society coding DNA sequence; *HGVSp*, Human Genome Variation Society protein sequence; *CM*, cardiomyopathy; *comp het*, compound heterozygous; *het*, heterozygous; *hom*, homozygous; *hemi*, hemizygous. ^a^PMID 34732400 [40]; ^b^doi-10.1111/bjd.21325 [41]; ^c^PMID 32396390 [42]; ^d^PMID 34212438 [43]. Further variant details are available in Table S12

Child no	Gene	HGVSc	HGVSp	Consequence	Zygosity	Diagnosis	Group	Diagnosis source	On CM panel	Notes
Probable research diagnosis										
3	ALPK3	c.3514del	p.Val1172Serfs*100	Frameshift	het	HCM	Complex	CM panel	Yes	In a solved patient
3	ALPK3	c.3485G > C	p.Gly1162Ala	Missense	het	HCM	Complex	CM panel	Yes	In a solved patient
63	ATAD3	NA	NA	Duplication	het	CM unspecified	Complex	diag	No	Published^a^
64	ATAD3	NA	NA	Duplication	het	CM unspecified	Complex	diag	No	Published^a^
65	DOLK	c.1372G > A	p.Gly458Ser	Missense	hom	DCM	Primary	Exomiser	Yes	
66	ELAC2	c.2009del	p.Cys670Serfs*14	Frameshift	comp het	DCM	Complex	Exomiser	Yes	Published^a^
66	ELAC2	c.2245C > T	p.His749Tyr	Missense	comp het	DCM	Complex	Exomiser	Yes	Published^a^
67	FHOD3	c.1646G > C	p.S549T	Splice region	het	HCM	Primary	CM panel	Yes	
68	FLNC	c.7756_7759del	p.Ser2586del	Inframe deletion	het	HCM	Primary	CM panel	Yes	
69	FLNC	c.5006_*576del	Exons 30–48	Deletion	het	DCM	Primary	SV, diag	Yes	
70	FLNC	c.6451G > A	p.Gly2151Ser	Missense	het	CM unspecified	Complex	CM panel	Yes	
71	JAK1	c.2666 T > C	p.Val889Ala	Missense	het	DCM	Complex	De novo	No	Published^b^; CM not known to be associated
72	MAP3K7	c.713G > A	p.Arg238Gln	Missense	het	DCM	Complex	De novo	No	
73	MTO1	c.938G > C	p.Arg313Pro	Missense	comp het	CM unspecified	Complex	Exomiser	No	
73	MTO1	c.1232C > T	p.Thr411Ile	Missense	comp het	CM unspecified	Complex	Exomiser	No	
74	MYBPC3	c.1927 + 600C > T	NA	Intronic	het	HCM	Primary	ClinVar	Yes	
75	MYBPC3	c.1927 + 600C > T	NA	Intronic	het	HCM	Primary	ClinVar	Yes	
76	MYBPC3	c.927-9G > A	NA	Intronic	het	HCM	Primary	ClinVar, diag	Yes	
77	MYBPC3	c.1224-21A > G	p.?	Intronic	het	HCM	Primary	ClinVar, diag	Yes	Published^c^
78	MYH7	c.4076G > A	p.Arg1359His	Missense	het	DCM	Primary	CM panel	Yes	
79	MYH7	c.2717A > G	p.Asp906Gly	Missense	het	HCM	Complex	CM panel	Yes	
80	MYH7	c.2682A > C	p.Glu894Asp	Missense	het	DCM	Primary	CM panel	Yes	
81	NAA15	c.174_177del	p.Cys58Trpfs*15	Frameshift	het	HCM	Primary	CM panel	Amber	
82	NF1	c.5305C > T	p.Arg1769*	Stop gained	het	DCM	Primary	ClinVar	Amber	Not robust disease gene
83	NPHP3	c.2805C > T	p.Gly935 =	Synonymous	hom	HCM	Complex	ClinVar	No	Published^d^; CM not known to be associated
84	PRKAG2	c.1148A > G	p.His383Arg	Missense	het	HCM	Primary	CM panel, GEL	Yes	
85	RYR2	c.11147A > G	p.Glu3716Gly	Missense	het	ARVC	Primary	De novo	Yes	Not robust disease gene
86	RYR2	c.169-198_273 + 823del	p.Asn57_Gly91del	Deletion	het	HCM	Primary	SV	Yes	Not robust disease gene
87	SCN5A	c.665G > A	p.Arg222Gln	Missense	het	DCM	Primary	ClinVar, diag	Yes	
88	SCN8A	c.3967G > A	p.Ala1323Thr	Missense	het	HCM	Primary	ClinVar, diag	No	CM not known to be associated
89	SCO2	c.323A > G	p.Asp108Gly	Missense	comp het	CM unspecified	Complex	Exomiser	Yes	Published^a^
89	SCO2	c.281 T > C	p.Leu94Pro	Missense	comp het	CM unspecified	Complex	Exomiser	Yes	Published^a^
90	TAB2	c.1354C > T	p.Arg452*	Stop gained	het	CM unspecified	Complex	CM panel, GEL	Yes	
91	TBX20	c.381-2A > C	NA	Splice acceptor	het	LVNC	Primary	CM panel	Yes	
92	TKFC	c.1628G > T	p.Arg543Ile	Missense	hom	DCM	Complex	ClinVar, diag	No	
93	TNNI3	c.535G > T	p.Glu179*	Stop gained NMD escaping	het	CM unspecified	Complex	CM panel	Yes	
Possible research diagnosis										
94	ALPK3	c.2043_2044delinsCT	p.Gln681_Glu682delinsHis*	Stop gained	het	DCM	Primary	ClinVar	Yes	Not robust disease gene
95	ALPK3	c.4772 + 1G > A	p.?	Splice donor	het	HCM	Primary	CM panel	Yes	
96	DMD	c.5739 + 404A > G	p.?	Intronic	hemi	DCM	Complex	CM panel	Yes	
97	FGFR3	c.667C > T	p.Arg223Cys	Missense	het	DCM	Complex	ClinVar	No	CM not known to be associated
98	FLNC	c.2551-55_2568del	NA	Deletion	het	CM unspecified	Complex	SV, diag	Yes	
99	FHOD3	c.1646 + 1_1646 + 4del	p.?	Splice donor	het	CM unspecified	Complex	CM panel	Yes	
100	KLHL24	c.1376 T > C	p.Ile459Thr	Missense	hom	HCM	Primary	CM panel	No	
101	LZTR1	c.2330 T > C	p.Leu777Pro	Missense	comp het	HCM	Primary	CM panel	Yes	
101	LZTR1	c.1735G > A	p.Val579Met	Missense	comp het	HCM	Primary	CM panel	Yes	
102	LZTR1	c.1616-8G > A	p.?	Splice region	hom	HCM	Complex	CM panel	Yes	
103	LZTR1	c.360C > A	p.His120Gln	Missense	het	HCM	Primary	De novo	Yes	
104	MYH7	c.1003G > T	p.Ala335Ser	Missense	het	HCM	Primary	CM panel	Yes	
105	NKX2-5	c.446A > C	p.Gln149Pro	Missense	het	DCM	Primary	CM panel	Yes	
106	RAF1	c.420C > A	p.Asn140Lys	Missense	het	HCM	Primary	CM panel	Yes	
107	RRAGC	c.936dup	p.Glu313Argfs*10	Frameshift	het	DCM	Primary	CM panel	Yes	
108	TPM1	c.461 T > C	p.Ile154Th	Missense	het	HCM	Complex	CM panel	Yes	
109	TPM1	c.82G > A	p.Asp28Asn	Missense	het	HCM	Primary	CM panel	Yes	
110	TREX1	c.45C > G	p.Ile15Met	Missense	hom	CM unspecified	Complex	Exomiser	No	
111	TTN	c.40927 + 166_42682 + 37del	p.?	Deletion	het	DCM	Primary	SV	Yes	

Potentially causative variants were predominantly identified in unsolved cases with Complex CM (19/49, 39%) or HCM (17/49, 35%), with a smaller proportion (10/49, 20%) identified in those with DCM. Family history was reported in 45% (22/49).

Twenty percent (10/49) were compound heterozygous or homozygous variants and 12% (6/49) were de novo dominant variants. For most, inheritance is unknown due to recruitment as singletons or duos. Structural variants and intronic and splice region variants predicted to impact splicing were found in 12/49 (24%) individuals.

#### Which advances have improved our yield?

Variants in several known genes were identified, including 8 variants in *MYH7* and *MYBPC3*. For *MYH7*, variant level evidence has grown over time allowing some previously classified variants of uncertain significance to be upgraded [44]. For *MYBPC3*, several intronic variants known to impact splicing have been implicated in HCM [42]. Most of these are deep intronic variants that would not have been returned for review initially but are now reported as P/LP in ClinVar. Using ClinVar helped to identify 11/49 (22%) potential diagnoses.

Using our broader candidate gene list, we identified 14/49 (29%) potential diagnoses in genes not on the original CM panels used by GEL (see Fig. 2). Six probands have variants in *FHOD3* and *FLNC*, genes more recently associated with CM. *FLNC* was updated to diagnostic grade (‘green’) for DCM on PanelApp in September 2019 and curated as definitively associated with DCM by ClinGen [45] in 2020 [46]; *FHOD3* was updated for HCM in PanelApp in December 2019. In *FHOD3*, we identified two variants affecting the same essential splice donor site of exon 12 reported previously in 3 unrelated families [16, 47].

Findings in rarer causes of CM include a heterozygous frameshift variant in *NAA15*, a gene associated with intellectual disability and congenital heart disease, but also found to cause HCM in 2 unrelated children [48]; a homozygous variant in *DOLK*, which is associated with congenital disorder of glycosylation type Im which includes a DCM phenotype; a homozygous variant in *TREX1* which is associated with Aicardi-Goutieres syndrome in which mouse models develop CM; and a homozygous variant in *KLHL24*, a gene known to be a rare recessive cause of HCM.

Using a gene agnostic de novo analysis, we identified four probands with LP de novo single nucleotide variants (SNVs). The genes were *RYR2*, *LZTR1*, *JAK1* and *MAP3K7*. In this PCM cohort, there are two probands with de novo missense variants in *MAP3K7*. Pathogenic variants in *MAP3K7* have been associated with cardiospondylocarpofacial syndrome (CSCF); phenotypically this can overlap with Noonan syndrome (NS). A study looking specifically at a cohort of patients with CSCF and *MAP3K7* variants observed 4/12 patients with CM (one HCM and three DCM) [49]. This gene is not routinely assessed in patients with either syndromic or isolated CM. *LZTR1* is associated with Noonan syndrome which includes HCM as part of the disease. There is limited evidence to support a relationship between *RYR2* and HCM, therefore further phenotype information will be required before a diagnosis can be established. *JAK1* is not known to be associated with CM.  

Updated analysis of SVs > 50 bp, filtered for rarity and our broad gene list, revealed deletions and duplications in 4 probands affecting *FLNC*, *TTN* and *RYR2*. A further two duplications affecting the *ATAD3* cluster were flagged through the diagnostic discovery pathway. Finally, other SNVs were prioritised using Exomiser [26] (see Table 4 and Additional file 1: Table S12 for full details).

Overall, by using our broader approach we identified variants in 34 unique genes. Seven of these 34 genes are not on the R135 CM panel but CM is a recognised feature of the associated disease, e.g. MTO1 related infantile hypertrophic cardiomyopathy and lactic acidosis (see Table 4). For a further three genes there is no association with CM reported (*JAK1*, *SCN8A* and *FGFR3*). These genes were identified from the de novo and ClinVar analysis and their known disease associations appear in keeping at least in part with the proband’s phenotype terms. However their contribution to CM is unknown therefore they may represent only partial diagnoses.

#### How does this analysis compare with other diseases that have been re-analysed in 100kgp

This re-analysis of the PCM cohort has identified a probable or possible diagnosis in 49 previously unsolved participants and has the potential to increase diagnostic yield by up to 18%. These results have been submitted to the diagnostic discovery pathway in GEL for further assessment before being sent to diagnostic laboratories for final variant classification.

Our outcomes are in keeping with those of other diseases that have been re-analysed. Hyder et al. demonstrated a similar diagnostic uplift from 14 to 29.8% in craniosynostosis patients and found a much higher success rate for syndromic presentations [50]. Similarly, Best et al. made a research diagnosis in an additional 19.3% of ciliopathy patients [51].

## Discussion

### The cardiomyopathy probands in 100KGP

In the 100KGP, 1918/90,190 (2%) of participants were recruited under CM or had ‘cardiomyopathy’ mentioned amongst their phenotypes; 1563/1918 are probands. There was an initial requirement for participants to have an affected family member at enrolment (except probands with HCM < 40 years). Severe, early onset disorders, such as PCM, are often sporadic because they negatively impact reproductive fitness. This understanding motivates the use of trio strategies to find de novo dominant and recessive genetic causes. It is therefore discordant to have a family history requirement while recruiting parent–child trios for gene discovery. Despite this, less than 50% of CM probands had a documented family history. We observed that individuals with a family history were significantly less likely to be included in a trio, suggesting that clinicians appropriately recruited trios for sporadic cases and were less likely to do so if a family history was present. Overall, 945/1563 (60.5%) CM probands were recruited as singletons, and only 215/1563 (14%) were recruited as full trios enabling de novo analysis. This number is driven by 147 paediatric trios (134 who have GEL de novo variant annotation).

CM probands were recruited across a huge breadth of categories, > 55 in total. While 34% of children with CM were recruited under non-CM categories, the range in recruitment categories was not exclusive to children. Nearly 10% of the adults were designated as Complex CM and were recruited under 34 non-CM disease categories, ranging from mitochondrial disorders to intellectual disability and early-onset dementia. This suggests both adult and paediatric CM patients are presenting to a variety of mainstream specialities outside of cardiology, which in turn has implications for timely diagnosis and management.

There were clear differences in the way adults and children were recruited, the proportion of syndromic presentations and the initial diagnostic yield. Interestingly, co-existing CHD was seen in nearly 12% of the paediatric cohort in comparison to 0.9% of the adults. The frequency seen in adults is more in keeping with background population estimates [52]. Kaski et al. have recently reported a high rate of co-existing CHD (16.5%) in their European paediatric cohort [13]. Most of the children with co-existing CHD in 100KGP remain genetically unsolved after our re-analysis suggesting that as well as known syndromes where both CM and CHD feature, there may be other, rarer genetic causes that are giving rise to both CHD and CM.

There is overlap between the genetic basis of adult and paediatric cardiomyopathy in 100KGP. Variants in 65 unique genes were identified by GEL’s initial analysis and the re-analysis of the paediatric unsolved probands; 18 of these overlapped between children and adults. In both age groups, variants were frequently identified in *MYH7*, *MYBPC3* and *TNNI3* for HCM and *RBM20* and *FLNC* for DCM.

### Re-analysis of paediatric cardiomyopathy probands can uplift diagnostic yield

Following GEL’s analysis which was completed in 2018, more than 80% of CM probands remained genetically unexplained. Since then, there has been an exponential rise in both access to sequencing and sharing of genomic data. New evidence regarding genes and variants associated with CM continues to emerge [53], as well as much larger population variant datasets [54] and improved tools for variant prioritisation. Re-assessing genomic data in the light of this new evidence is necessary and can be very effective [55]. We focused on the paediatric cohort hypothesising that there may be a higher frequency of unidentified monogenic causes in those with early onset disease. GEL reported 62 paediatric probands as solved or partially solved. We identified a probable or possible diagnosis fully or partially contributing to the phenotype for an additional 49 genetically unsolved probands. This has the potential to uplift diagnostic yield to up to 40%. Work on other disease cohorts within 100KGP has had similar results emphasising the importance of iterative re-analysis.

In total, potential diagnoses were made in 111 paediatric CM probands (this includes both GEL’s initial analysis and our re-analysis). Intronic or splice region variants predicted to impact splicing, or structural variants were identified in 12/111 (11%); variant types more easily identified by genome sequencing. De novo variants were identified in 19/111 (17%). The proportion of diagnoses that were de novo may be an underestimate given only half of all the PCM probands had trio data available. Fourteen probands (7 with a Hot VUS and 7 with a VUS) where inheritance was unknown could have had variants reclassified with confirmation of de novo status. Some studies have found a higher proportion of diagnoses were de novo [15, 56]. In their study of 66 children with CM in Finland, Vasilescu et al. report 46% of their findings as de novo, however, only patients with severe, early onset disease, presenting for transplant assessment were included. The lower frequencies seen here could therefore reflect the range in age and presentation of this cohort, in addition to the initial eligibility requirement for family history.

Despite this being a discovery cohort, several established CM genes have been implicated in the diagnosis for more than one paediatric proband, including *MYH7*, *MYBPC3*, *TNNI3*, *TNNT2*, *RBM20*, *FLNC*, *SCN5A*, *LZTR1*, *RAF1*, *ALMS1* and *NKX2-5*. However, substantial genetic heterogeneity means that most other established CM genes only contribute to a single diagnosis. It is noteworthy that 2 probands have de novo variants in *MAP3K7*, a gene associated with cardiospondylocarpofacial syndrome but not known to cause primary CM. In addition, 2 probands presenting with features of mitochondrial disease and CM were found to have *ATAD3* cluster duplications. These were established as pathogenic after GEL’s initial analysis [40, 57].

All the methods employed in our re-analysis contributed to identifying new potential diagnoses. The main uplift came from analysing genes not included in previous panels and incorporating ClinVar data. Many of our findings are in genes considered putative CM genes, however, the available evidence supporting these gene-disease associations varies significantly, which impacted our classification of certain variants.

We note that 133 genes are present in other CM panels but are not included on the current UK R135 CM panel ‘green’ list (see Fig. 2). This highlights the variability in clinical testing for paediatric CM. Even within the UK, there is significant variability with some laboratories only analysing PanelApp ‘green’ (genes with the highest evidence) while others also include ‘amber’ (genes with moderate evidence). In this study, we identified several genes where CM is a known feature of the associated disease but these genes are not currently included on the R135 CM panel. It would be reasonable to add these genes to a syndromic CM panel. Specifically, for *MAP3K7*, CM can be a principle, if not the presenting, feature of the disease so this gene should be routinely evaluated. Overall, more work is needed to curate a comprehensive list of genes strongly associated with early onset CM. Through both GEL and our own re-analysis, several P/LP variants were found in genes associated with part of the patient’s phenotype, but not known to be associated with CM.

Further work will be required to determine whether there is another explanation for the CM in these cases or whether these could represent phenotype expansion.

### The remaining unsolved probands—where are the missing diagnoses?

Despite our re-analysis, there are still 161/272 PCM probands without a potential genetic diagnosis and still more where the variant identified may not fully explain the phenotype. Complex CM makes up a greater proportion of solved cases than unsolved, *p* = 0.045. However, there are no other statistically significant differences between the solved and unsolved groups in terms of demographics including family history, age at consent, ancestry and diagnosis. There is continuing debate about where missing diagnoses lie. Non-genetic causes, oligogenic inheritance, non-coding variants, structural variants, genes not yet known to be associated with monogenic CM and VUS not meeting ACMG criteria could all be contributing. In this work, we would have been able to classify more indeterminate variants if there was more evidence to support some of the rarer gene disease associations and if trio data had been available for more participants.

### Large-scale human genomics studies have limitations (see Table 5)

**Table 5:** Avoiding hidden pitfalls to optimise using 100KGP data for cardiovascular research

**1. Initial eligibility criteria for prior testing of known genes were relaxed **
This occurred throughout the time course of the project for cardiomyopathies. Therefore, while the cohort is depleted for known genetic diagnoses, some recruited probands do have pathogenic/likely pathogenic variants in known disease genes.
**2. Initial eligibility criteria for family history requirements changed**
For cardiomyopathies, family history requirements were relaxed during the course of the project. Conversely for a minority of disease categories, such as renal and urinary tract disorders, clinical criteria were tightened.
**3. Recorded age of onset is unreliable for early onset diseases**
Where age of onset was not entered GEL assigned a value of zero making recorded age of onset unreliable, particularly for early onset diseases, i.e. congenital onset is indistinguishable from missing data.
**4. The majority were recruited as singletons limiting scope for de novo analysis**
Only 14% of the cardiomyopathy cohorts were recruited as trios (proband, mother and father), which limits the scope for *de novo *analysis. The proportion of probands recruited as trios is likely to vary across different disease categories and be impacted by age of onset of disease (for cardiomyopathies, children were more likely to be recruited as trios compared to adults), and by family history requirement (for cardiomyopathies, probands with a positive family history were less likely to be recruited as trios).
**5. Phenotype recording varies in detail **
For cardiomyopathies, it often just includes 1 or 2 features key to eligibility and is in the form of human phenotype ontology (HPO) terms. Objective measures of disease such as ejection fraction are not currently available. Deep phenotype data which would allow reverse phenotyping is typically unavailable.

Eligibility criteria for CM in 100KGP was initially strict and included prior genetic testing and a positive family history. However, these mandates were later relaxed to accelerate recruitment, and so that inequalities in access to routine diagnostic testing in the NHS at that time did not disqualify some individuals from participating in research, and thereby compound inequality. This has resulted in a ‘discovery’ cohort contaminated with findings in known genes. For example, variants in *MYBPC3*, *MYH7* and *TTN* account for 72/146 (49%) of solved adult CM probands. Pathogenic variants in these genes should have been identified by testing prior to enrolment. It is therefore difficult to draw conclusions about diagnostic yield and the contributions of different genotypes that are generalisable to other CM cohorts. The proportion of *MYBPC3* variants we observe in the 100KGP HCM cohort is depleted compared to what we would expect in an unselected HCM disease cohort. We also see depletion of *TTN* truncating variants in DCM, however less so, reflecting that clinical testing was offered less often to patients with DCM at the time of 100KGP. For the 100KGP paediatric CM cohort, we can conclude it is a large mixed cohort of all types of CM including syndromic forms, and clearly many participants had severe disease (14% have died, the majority before the age of 5). This is higher than the 11% reported in an Australian study [14] and much higher than the 3.3% reported in a major European study [13], although the latter excluded children under the age of 1.

Defining age of onset meaningfully in 100KGP is challenging since an age of zero was assigned for missing values. We distinguished adults and children using a variety of parameters recognising that there would still be a degree of inaccuracy. This meant we were unable to reliably observe expected differences in diagnostic yield and genetic architecture by age [10] within the paediatric cohort, in particular to compare infant onset to later onset paediatric disease. Despite this limitation, it is still possible to see clear differences in the characteristics of the adult and paediatric cohorts, including co-existing CHD rates, syndromic presentations and genetic causes. Contacting recruiting clinicians to clarify participant age and gather more phenotype information is possible through a clinical collaboration request in the RE. However, previous researchers have reported a poor response rate, particularly as time has elapsed since recruitment [58]. This is clearly an issue that goes beyond the CM cohort.

In large multi-centre studies, phenotype recording will vary in its accuracy and detail and at times may even be misleading. CM patients can present through multiple different specialities and the HPO terms reported may be influenced by a clinician’s specialty. This is demonstrated throughout the CM dataset. The number of HPO terms reported ranged from 1 to 43 with a median of 3 for Primary CM participants and 9 for Complex CM. There are instances where participants were recruited under one type of CM and then other types were listed in the HPO terms making it extremely difficult to accurately define disease groups. Objective measures of disease such as cardiovascular magnetic resonance parameters are currently not available. In a large national cohort, obtaining standardised data on a wide variety of diagnostic modalities is challenging, both due to heterogeneity of investigative pathways and due to the time required from healthcare staff to collect and submit the data. However, there is work underway to enrich the 100KGP dataset with these primary (unstructured) datasets for some conditions. In summary, the lack of reliability of reported phenotypes in 100KGP means caution is required when assessing novel findings.

### Other limitations

Significant genetic and allelic heterogeneity in PCM make it particularly challenging to know if a variant is the actual cause of the disease. In our unsolved analysis, we have included information justifying our current variant classification but recognise that these classifications are not static and new evidence can come to light at any time (see Additional file 1: Table S12). These variants still need to be assessed by the participant’s clinician and validated in a clinical laboratory before a diagnosis can be established. Mitochondrial DNA variants were not assessed as part of this re-analysis and these may provide additional insights in future work.

## Conclusions

The huge growth in access to sequencing and sharing of genomic data is providing ever bigger cohorts for use in discovery and replication in genetic research. These large-scale human genomics studies are a powerful resource, enabling collaboration and opportunities for researchers.

Here we have described and compared the clinical and molecular characteristics of paediatric and adult CM probands in 100KGP, an important national cohort. Notably, this cohort has 272 PCM probands with genome sequencing data, a significant contribution to the overall number of patients available for research for this rare condition. We demonstrate the benefit of re-analysing their genomic data, achieving a potential diagnostic uplift of 18%. The value of iterative re-analysis has been shown in other disease areas as well. However, we recognise that incorporating this in routine clinical practice presents considerable practical challenges, and currently it may largely be through research efforts that patients have their data re-evaluated.

While most potentially causative variants were found in coding regions, 11% (structural variants and intronic variants impacting splicing) would be more readily identified by genome sequencing. Clearly, in a disease cohort where many are presenting with early onset sporadic disease, access to trio data enables upfront confirmation and prioritisation of de novo variants which in this situation may well be causal. Overall our findings support the use of genome sequencing (GS) over targeted panels and exome sequencing for paediatric CM testing where it is possible. In the NHS, GS is already standard practice for paediatric and syndromic CM. Importantly however, the scope of analyses varies considerably between laboratories and is often restricted to a virtual panel. Better curation of gene disease relationships for paediatric and syndromic CM and ongoing sharing of clinical and research findings will help to standardise clinical panels and improve future variant identification.

## Supplementary Information

Additional file 1: All supplementary tables. Table S1 Candidate gene list used as part of the re-analysis of paediatric cardiomyopathy probands. Table S2 List of genes included in different paediatric or syndromic cardiomyopathy panels. Table S3 List of genes associated with cardiomyopathy identified from literature. Table S4 List of genes in version 1 of the UK PanelApp cardiomyopathy panels. Table S5 Filters used for de novo variant annotations in 100,000 Genomes Project taken from Genomics England Research Documentation, https://re-docs.genomicsengland.co.uk/de_novo_data/. Table S6 Demographic and clinical details of the paediatric and adult cardiomyopathy cohorts when stringent criteria are used to define children (age at consent ≤ 16 years). Table S7 Family structures at enrolment for the paediatric and adult cardiomyopathy cohorts when stringent criteria are used to define children (age at consent ≤ 16 years). Table S8 Number of paediatric and adult cardiomyopathy probands by disease recruitment category in the 100,000 Genome Project and the number and percentage of solved probands, using stringent criteria to define children. Table S9 Recruitment categories used for those paediatric and adult probands (Complex CM probands) not recruited under a cardiomyopathy specific category. Table S10 Variant details for genetically‘solved’ paediatric cardiomyopathy probands following GEL’s initial analysis. Table S11 Variant details for genetically ‘solved’ adult cardiomyopathy probands following GEL’s initial analysis. Table S12 Variant details for paediatric cardiomyopathy probands following a re-analysis.

## Data Availability

All datasets generated during this study are included in this published article and Additional file 1. Research on the de-identified patient data used in this publication can be carried out in the Genomics England Research Environment subject to a collaborative agreement that adheres to patient led governance. All interested readers will be able to access the data in the same manner that the authors accessed the data. For more information about accessing the data, interested readers may contact research-network@genomicsengland.co.uk or access the relevant information on the Genomics England website: https://www.genomicsengland.co.uk/research.

## Abbreviations

100KGP: The 100,000 Genomes Project
ACM: Arrhythmogenic cardiomyopathy
ARVC: Arrhythmogenic right ventricular cardiomyopathy
ClinGen: The Clinical Genome Resource
CM: Cardiomyopathy
CSCF: Cardiospondylocarpofacial syndrome
DCM: Dilated cardiomyopathy
GEL: Genomics England
GMC: Genomic Medicine Centre
GMS: Genome Medicine Service
GS: Genome sequencing
HCM: Hypertrophic cardiomyopathy
HES: Hospital Episode Statistics
HPO: Human phenotype ontology
LP: Likely pathogenic
LVH: Left ventricular hypertrophy
LVNC: Left ventricular non-compaction
NHS: National Health Service
NS: Noonan syndrome
P: Pathogenic
PAV: Protein altering variant
PCM: Paediatric cardiomyopathy
R135 CM panel: NHS Genomic Medicine Service paediatric or syndromic cardiomyopathy panel (R135 v3.44)
RCM: Restrictive cardiomyopathy
RE: Research environment
SNV: Single nucleotide variant
SV: Structural variant
VEP: Variant Effect Predictor
VUS: Variant of uncertain significance
